# TeloPIN: a database of telomeric proteins interaction network in mammalian cells

**DOI:** 10.1093/database/bav018

**Published:** 2015-03-18

**Authors:** Zhenhua Luo, Zhiming Dai, Xiaowei Xie, Xuyang Feng, Dan Liu, Zhou Songyang, Yuanyan Xiong

**Affiliations:** ^1^Key Laboratory of Gene Engineering of the Ministry of Education and State Key Laboratory of Biocontrol, School of Life Sciences, Sun Yat-Sen University, Guangzhou 510006, China, ^2^Department of Electronics and Communication Engineering, School of Information Science and Technology, Sun Yat-Sen University, Guangzhou, China; ^3^SYSU-CMU Shunde International Joint Research Institute (JRI) Shunde, Guangdong, China; ^4^Cell-Based Assay Screening Core, ^5^Dan L. Duncan Cancer Center, ^6^Verna and Marrs Mclean Department of Biochemistry and Molecular Biology, Baylor College of Medicine, One Baylor Plaza, Houston, TX 77030, USA, and ^7^Key Laboratory of Reproductive Medicine of Guangdong Province, Guangzhou, China

## Abstract

Interaction network surrounding telomeres has been intensively studied during the past two decades. However, no specific resource by integrating telomere interaction information data is currently available. To facilitate the understanding of the molecular interaction network by which telomeres are associated with biological process and diseases, we have developed TeloPIN (Telomeric Proteins Interaction Network) database (http://songyanglab.sysu.edu.cn/telopin/), a novel database that points to provide comprehensive information on protein–protein, protein–DNA and protein–RNA interaction of telomeres. TeloPIN database contains four types of interaction data, including (i) protein–protein interaction (PPI) data, (ii) telomeric proteins ChIP-seq data, (iii) telomere-associated proteins data and (iv) telomeric repeat-containing RNAs (TERRA)-interacting proteins data. By analyzing these four types of interaction data, we found that 358 and 199 proteins have more than one type of interaction information in human and mouse cells, respectively. We also developed table browser and TeloChIP genome browser to help researchers with better integrated visualization of interaction data from different studies. The current release of TeloPIN database includes 1111 PPI, eight telomeric protein ChIP-seq data sets, 1391 telomere-associated proteins and 183 TERRA-interacting proteins from 92 independent studies in mammalian cells. The interaction information provided by TeloPIN database will greatly expand our knowledge of telomeric proteins interaction network.

**Database URL:** TeloPIN database address is http://songyanglab.sysu.edu.cn/telopin. TeloPIN database is freely available to non-commercial use.

## Introduction

Mammalian telomeres are specialnucleo protein structures at the ends of chromosomes (1–7). Telomeres are extended primarily through the action of the telomerase (which consists of the reverse transcriptase TERT and the RNA template hTR). In addition, a large array of telomeric proteins regulates telomere length and protects telomere ends from being recognized as DNA breaks. These proteins together form the vast network of proteins at the telomeres that ensure genome stability and integrity (8–10). Accumulating evidence has clearly linked telomere maintenance to aging, stem cell exhaustion and diseases such as cancer as well as premature aging syndromes including dyskeratosiscongenita (DC), Hutchinson Gilford Progeria Syndrome and idiopathic pulmonary fibrosis (11–15). For example, mutations of telomerase subunits and telomere proteins (e.g. TERT, dyskerin and TIN2) have been identified in patients with DC and aplastic anemia (16, 17). It has been demonstrated that these mutations can alter protein–protein interactions (PPIs), thus impacting the signal transduction pathways within the telomere interaction network. As a result, understanding how telomeric proteins interact with each other on telomeres will not only prove essential to elucidate the mechanisms of telomere maintenance, but also facilitate our research into treating diseases such as DC and aplastic anemia.

Mammalian telomeres consist of (TTAGGG)*n* double-stranded DNA repeats sequences and terminate in single-stranded 3’-overhangs that invade into the double-stranded region to form the so-called T loop structure (18–21). The telomere interaction network is anchored by a six protein complex, also called telosome/shelterin, and is composed of TRF1, TRF2, TIN2, TPP1, POT1 and RAP1 (8, 22). Three of these proteins can directly interact with telomere DNA. TRF1 and TRF2 interact with the telomere duplex region, while POT1 binds to telomere single-stranded DNA (ssDNA) (23–26). Although TPP1 itself has no demonstrable DNA binding activity, it enhances the interaction of POT1 with telomere ssDNA (27). RAP1 is recruited to telomeres through its interaction with TRF2 (28), andTIN2 is recruited to by binding to TRF1 (29). TIN2 also functions as a bridge connecting TRF1/TRF2 with TPP1 (8). The telosome complex regulates telomerase recruitment and telomere length, and plays an important role in DNA damage response at telomeres (1, 2, 22, 30). For example, TPP1 can directly interact with TERT and recruit the telomerase to telomeres (27, 30–32). Furthermore, the telosome interacts with other proteins, thereby assembling the network of interacting proteins on telomeres. For example, we have shown that TRF2 is an important node in the interaction network (33). It can interact with TIN2 and RAP1 to telomeres, also with other proteins including PNTUS and MCPH1, to fulfill telomere regulation (33). In addition, some proteins with DNA binding activities (not specific for telomere DNA sequence) can directly bind to telomere DNA. For instance, we have shown that TAH1/HMBOX1/HOT1 directly binds to telomeric double-stranded DNA and become a part of the interaction network (34, 35).

In addition to telomere DNA and telomeric proteins, another factor in the telomere interaction network is RNA. Telomeres are also constitutively transcribed into telomeric repeat-containing RNAs (TERRA), which are long non-coding RNAs with variable length (36). TERRA localizes to telomeres and is transcribed from several subtelomeric loci toward chromosome ends (36). TERRA can interact with a number of telomeric proteins (e.g. TRF1 and TRF2) and has been implicated in telomere heterochromatin maintenance, telomerase regulation and telomere capping (37–39).

Recent advances in biotechnology have tremendously accelerated our progress in elucidating the telomeric interaction network. Our group carried out a genome-wide YFP fluorescence complementation screen for regulators of telomeres and identified over 300 proteins that could interact with the telosome/shelterin complex (40). This screen offered a large set of candidate proteins that were never linked to telomere maintenance before. The powerful approach termed proteomics of isolated chromatin segments (PICh) developed by Dejardin and Kingston (41) has enabled the study of novel telomeric proteins that associate with telomere DNA. Recently, Bartocci *et al.* (42) employed the PICh approach to define changes in telomeric chromatin composition between functional (TRF2-proficient) and dysfunctional (TRF2-deficient) telomeres, revealing Ring1b as an important regulator in NHEJ-mediated chromosome fusions. For quantitative assessment of differences in telomere protein composition in cells with different telomere states, Grolimund *et al*. (43) developed a quantitative telomeric chromatin isolation protocol (QTIP) that combines chromatin isolation with the stable isotope labeling by amino acids in cell culture (SILAC) methodology (43). PICh and QTIP provide complementary information regarding protein interaction with the telomere DNA. Moreover, our group and others have mapped the genome-wide distribution of telomeric proteins TRF1, TRF2 and RAP1 in human and mouse cells by Chromatin immunoprecipitation coupled with massively parallel DNA sequencing (ChIP-seq) (44–47), and uncovered genome-wide extra-telomeric sites occupied by TRF1, TRF2 and RAP1 (44–46). In fact, a series of biochemical and genetic studies have underlined the extra-telomeric function for TRF2 and RAP1 in cancer development and obesity, respectively (48–50). And biotinylated RNA oligonucleotides were successfully used to identify several TERRA-associated proteins, including TRF1, TRF2, heterochromatin protein 1 and heterogeneous nuclear ribonucleo protein family (6, 37). In particular, in the study by Scheibe *et al.* (51) that relied on SILAC-based quantitative mass spectrometry (MS), 115 proteins were found to bind TERRA, vastly expanding the telomere DNA–RNA–protein interaction network and deepening our understanding of TERRA regulation.

**Figure 1.**
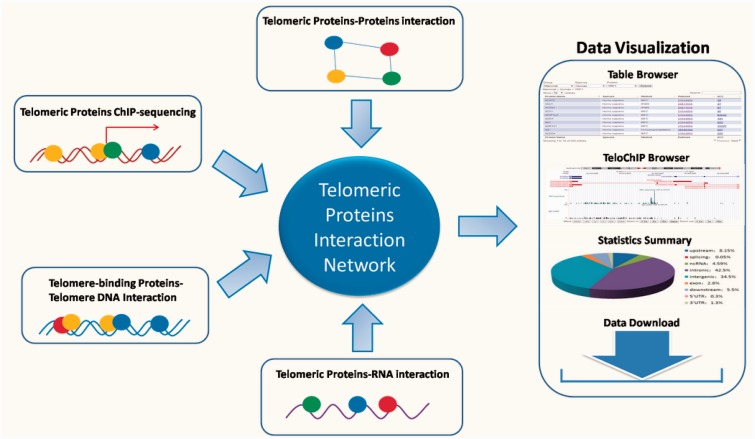
An overview of TeloPIN framework. 1111 Protein–Protein Interaction (PPI), 8 ChIP-seq data, 1390 telomere-binding proteins and 183 TERRA-interacting proteins were compiled and analyzed. All results were deposited and can be downloaded from MySQL databases and displayed in table or TeloChIP browser.

Despite the rapid accumulation of information regarding the telomeric interaction network, there remains a lack of a database that enables rapid access and integrated analysis of the interaction data. Such a database should not only prove essential to comprehensively understand signaling pathways at the telomeres, but also offer valuable insight into potential mechanisms for disease development. In this study, we describe our development of the TeloPIN database that aims to provide comprehensive information on telomeric protein–protein, protein–DNA and protein–RNA interaction in mammalian cells (Figure 1). All interaction information is summarized in Table 1 and Supplementary Table 1.

**Table 1.** Summary of TeloPIN datasets

Species	Telomeric protein–protein interaction	Telomeric protein–DNA interaction		TERRA-binding Proteins	Telomeric protein–DNA–RNA interaction	Studies
		Telomere-binding Proteins	ChIP-seq peak			
Homo sapiens	1095	516	31 100	142	358	86
Musmusculus	16	874	7580	41	199	6

Statistics show the numbers of Telomeric PPI, Telomere-binding proteins, Telomeric proteins ChIP-seq peak and TERRA-binding proteins

## Construction and content

### Assembling the telomeric protein–protein interaction dataset

**Figure 2.**
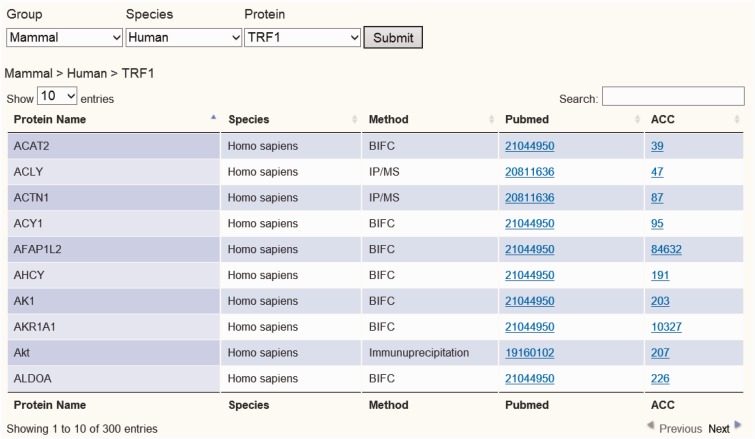
Screen shot depicting the Table Browser. Table Browser provides interactions information of telomeric protein–protein, protein–DNA and protein–RNA interaction, including protein name, species, method, Pubmed link and accession number link, etc.

To establish the telomeric interaction network database, we started by collecting protein interaction information regarding the six core telomere proteins and searched NCBI Pubmed using keywords for human and mouse TRF1, TRF2, RAP1, TPP1, POT1 and TIN2 (as well as alias) to obtain their experimentally identified interacting partners. All search results were crosschecked to avoid any wrong assignment because of different proteins sharing the same names. A total of 1111 interactions were found for the six proteins in both human and mouse. These interactions had been identified using a variety of biochemical and genetic methods, including yeast two-hybrid (Y2H), immunoprecipitation/MS (IP/MS), Bimolecular Fluorescence Complementation (BiFC) assay and pull-down assay. These data have been integrated so that they can be viewed in different configurations, either across all six proteins or for one of the six proteins. We assembled all the data as a whole table or individual table according to six telomeric proteins. Users can view the data either in whole or just for a particular telomeric protein. A Pubmed link is also provided for each interacting partner so that the accession number as well as the original interaction studies can be easily accessed (Figure 2 and Supplementary Figure 1).

### Assembling the protein–telomere DNA Interaction dataset

**Figure 3.**
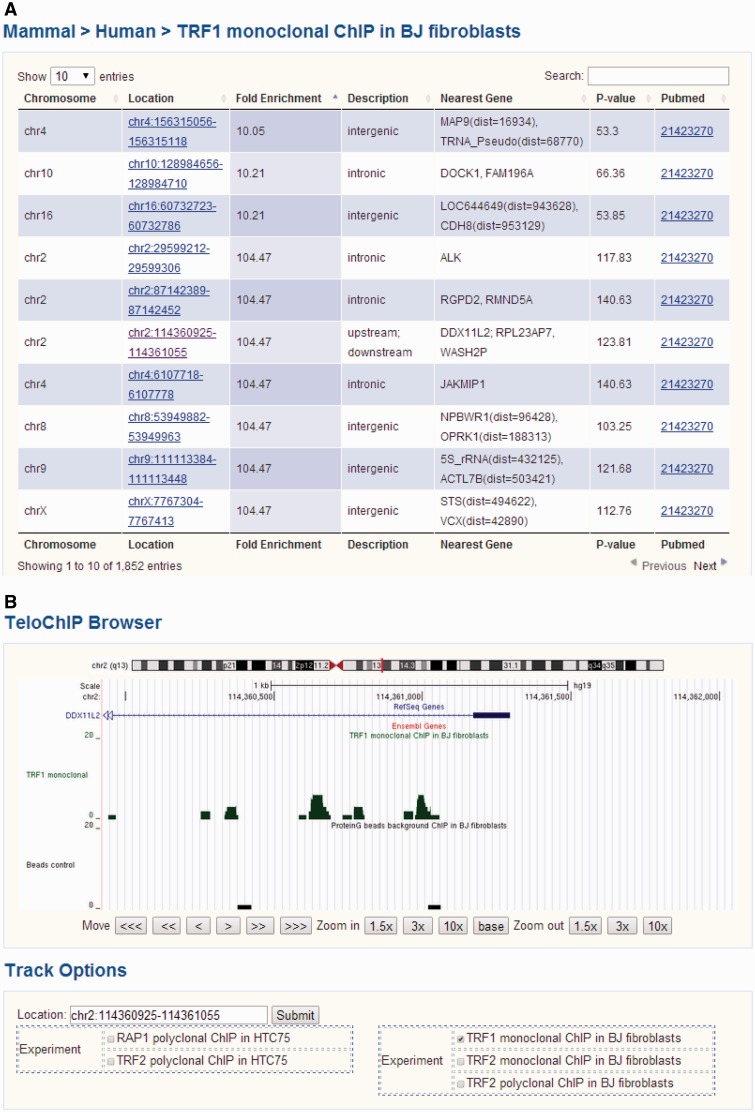
Screen shot depicting the Table Browser and TeloChIP Browser for telomeric protein ChIP data. **(A)** Table Browser provides telomeric protein ChIP-seq peak information, including chromosome location, fold enrichment, annotation, Pubmed link, etc. **(B)** TeloChIP Browser provides an integrated view of ChIP-seq peak location with chromosome location, RefSeq genes, Ensembl gene.

We divided protein–telomere DNA interaction data based on whether the particular proteins were known telomere-associated proteins or identified through telomere ChIP studies. For the latter, ChIP-seq results for TRF1, TRF2 and RAP1 were gathered from the NCBI Pubmed and GEO databases (Supplementary Table 1) (44–47). We aligned the ChIP-seq short reads to the human (hg19 release, UCSC) or mouse (mm10 release, UCSC) genomes using Bowtie 2, allowing for a maximum of two mismatches (52). For reads that were longer than 35 bp and could not be mapped to the genome, only the first 35 bp were used for alignment. All short reads that were uniquely aligned were used for further analysis. The program MACS (version 1.4.2) was used for peak detection under default settings (53). Annotation of the identified peaks was carried out using human genomic sequences (hg19) and mouse genomic sequences (mm10). Gene loci information was based on UCSC exon locus annotation hg19 (http://hgdownload.cse.ucsc.edu/goldenPath/hg19/database/), mm10 (http://hgdownload.cse.ucsc.edu/goldenPath/mm10/database/) and BioMart (http://www.biomart.org/). For exon annotation, the longest transcript was considered for each gene. Regions within 5 kb up or downstream of annotated transcripts were referenced as 5’UTR or 3’UTR, respectively. To display all of the ChIP-seq data, we developed the genome browser TeloChIP to enhance the visualization and comparison of ChIP-seq data across different studies (Figure 3).

Telomere-associated proteins came from studies using the PICh and QTIP methods. PICh is a reverse ChIP technique that relies on Locked Nucleic Acids containing oligonucleotides and mass spectrometric analysis to isolate and identify proteins targeted to specific genomic loci (33), and has been applied to study proteins–telomere DNA interactions in different cell types with different telomere states (41, 42). These studies identified 208 and 189 proteins to associate with telomeres in HeLa 1.2.11 and Wi38-VA13 cells, respectively (41), and 787 and 559 proteins in MEFs with functional (TRF2-proficient) vs. dysfunctional (TRF2-deficient) telomeres (42). In the study using MEFs, 471 proteins were found to associate with telomeres regardless of telomere status (42). The technique QTIP utilizes SILAC coupled with mass spectrometric analysis to compare and identify quantitative differences between telomere proteins (34). A total of 258 telomere-associated proteins were identified by QTIPin HeLa cells (43). Our analysis of both PICh and QTIP data from human cells revealed 38 proteins that had been recovered by both techniques. These shared targets may have a higher affinity for telomere DNA or they may localize to telomeres at higher concentrations (Supplementary Table 2). Furthermore, 23 out of the 38 proteins appeared to associate with telomeres both in human and mouse cells, suggesting that their activities on telomeres may be evolutionarily conserved (Supplementary Table 2).

### Assembling the telomeric protein–RNA interaction dataset

To integrate telomere protein–RNA interaction data into the interaction network, we analyzed the results from three large-scale proteomic studies that focused on telomeric proteins that could interact with the telomere transcript TERRA (6, 37, 51). A total of 183 TERRA-interacting proteins were uncovered in mammalian cells. Of these, 142 and 41 proteins were, respectively, identified from human and mouse cells. Interestingly, only four proteins were shared by human and mouse cells, including FXR1, hnRNPA1, hnRNPM and TJP1. These observations suggest complex roles for TERRA in telomere regulation and indicate distinct pathways between human and mouse cells.

### Identifying telomeric protein–DNA–RNA Interactions

The integration of various protein–protein, protein–DNA and protein–RNA interactions then enabled us to further process the data and identified those proteins that may be capable of tertiary interactions. The protein–DNA–RNA interaction table illustrates this result (Supplementary Figure 5). Interestingly, 358 and 199 proteins were found in more than one type of interaction in human and mouse cells, respectively. These findings demonstrate the power of such analyses and offer unique insight into the telomere regulatory network of interactions.

### Statistics summary and data download

We named our database TeloPIN. In addition to displaying the interaction information for each protein, TeloPIN also provides statistical summaries and data download capabilities. For example, users can download all interaction data in the ‘Statistics Summary and Data Download’ page (Supplementary Figure 6). The statistics summary contains the number of interacting proteins and the distribution of telomeric protein ChIP-seq data. All of the detailed information of interaction data including ChIP-seq annotation can be downloaded through TeloPIN (Supplementary Figure 6).

## Utility

### Table browser for protein–protein, protein–DNA and protein–RNA data and their regulatory relationships

Users can easily survey all interaction data through the TeloPINtable browser by entering gene symbol, gene ID and species or by simply browsing through specific telomeric proteins or telomeric RNA TERRA (Figure 2 and Supplementary Figures 1, 3 and 4). After each query, TeloPIN returns data in a table format detailing protein name, species/Cell line, method and Pubmed and accession links (Figure 2 and Supplementary Figures 1, 3 and 4). The Table browser interactively links the specific protein to Pubmed and NCBI databases for further references. For telomeric protein ChIP-seq data, the user can choose a specific ChIP-seq dataset and obtain a table with information on ChIP-seq peak location, fold enrichment, location annotation and Pubmed links (Figure 3 and Supplementary Figure 2). Furthermore, a cross-analysis table is provided in the Proteins–DNA–RNA section for integrating all interaction data to identify interaction hubs (Supplementary Figure 5). In this section, proteins with more than one type of interaction are listed in the table.

### Using the TeloChIP Browser for comparative analysis of telomeric proteins–gene regulatory relationships

To better visualize ChIP-seq data and peak location, TeloChIP browser was developed (Figure 3 and Supplementary Figure 2). TeloChIP is launched by clicking the link in the ‘Location’ column within the Telomeric Protein ChIP table browser (Figure 3 and Supplementary Figure 2). TeloChIP provides an integrated view of chromosome, RefSeq genes and Ensembl gene and peak location (Figure 3 and Supplementary Figure 2). Users can shrink or extend the width of the displayed coordinate range by zooming in and out. Importantly, ChIP-seq data of different telomeric proteins or studies can be displayed in parallel. When users click on the track options, a multiple-alignment trace view is launched that displays all traces that are of interest as well as the related control groups, enabling a more comprehensive comparison of data from different studies (Figure 3 and Supplementary Figure 2).

## Discussion

In this study, we have developed the TeloPIN database for the collection, visualization, identification and analysis of telomeric protein–protein, protein–DNA and protein–RNA interaction data. TeloPIN serves as a platform for assembling telomeric protein interaction data and to assist researchers in the reinvestigation of telomere maintenance and dysfunction. Compared with resources such as the Telomerase Database (54), TeloPIN has several distinct features. While the Telomerase Database contains information about the telomerase, TeloPIN for the first time offers a resource where comprehensive information on experimentally validated telomere protein–protein, protein–DNA and protein–RNA interactions can be obtained through a user-friendly website, and an easily accessible search tool for data visualization and analysis. In particular, we developed TeloChIPto better visualize ChIP-seq data, which enables seamless browsing throughout the genome and quick sampling of large sets of data. More importantly, users can compare different ChIP-seq datasets for the same protein and obtain ChIP peak information on a specific locus. TeloPIN also allowed us to identify proteins that have more than one type of interaction, information that may be particularly valuable in our understanding of regulatory interactions.

Our initial analysis reveals that some interacting partners of the core telomeric proteins can bind to telomere DNA or TERRA (directly or indirectly), suggesting that these proteins may play an essential role in telomere regulation. For example, previous study has shown that poly(ADP-ribose)polymerase 1 (PARP1), an interacting protein of RAP1 and TRF2, functions as regulator of telomere length and end protection (55, 56). In our protein–DNA–RNA analysis, PARP1 also binds to telomere DNA and TERRA. Whether PARP1 regulates TERRA stability, localization and/or modification remains to be investigated. Our analysis also indicates that some proteins can interact with both telomere DNA and TERRA, implying that these proteins may bridge TERRA association with telomere DNA or that their concentration on telomeres may be controlled by TERRA. In support of these observations, previous studies have identified a RPA-to-POT1 switch on telomeres that is orchestrated by TERRA and hnRNPA1 (57). Integrated analysis of different datasets also revealed that core telomeric proteins such as TRF2 can target to gene loci of its interacting partners (e.g. PML), raising the possibility that TRF2 may regulate the dynamics of protein–protein interaction networks and the integrity of telomeres through transcriptional control. Interestingly, a number of proteins involved in signaling transduction and metabolism appear to interact with core telomeric proteins and/or TERRA, thereby linking metabolic control and specific signaling pathways to telomere maintenance. For example, the subunit of 3-methylcrotonyl-CoA carboxylaseMCCC2 interacts with TRF2 and TERRA. Mutations of MCCC2 have been shown to be associated with the autosomal recessive disorder of leucine catabolism termed 3-MethyIcrotonylglycinuria (58). The MCCC2–TRF2–TERRA interaction may indicate telomere dysfunction in this metabolic disease. Recently, telomeric proteins have been found to play extra-telomeric roles in biological processes and diseases including NF-κB signaling (59), obesity regulation (49, 50), NK cell immunity (48) and neural tumor/stem cell fate control (60). Taken together, TeloPIN should offer unprecedented opportunities to systematically examine and analyze available data and identify new areas for further investigation.

Thus, we expected that the visualizable, integrative and systematic interaction information provides by TeloPIN will also aid future experimental studies and hypothesis test in diverse fields. In summary, the interaction data provided by TeloPIN database will help researchers for data analysis, interpretation and discover. Further works will focus on elucidating the functional protein interaction and transcription regulation by telomeric proteins at different cell types/tissues and conditions.

Investigating on the interaction network among these three molecules is critical for our better understanding of molecule biology and disease processes. The interaction network of telomeric proteins represents the molecular machinery that regulates telomere function. The establishment of interaction network also enables the integration of the intricate regulatory circuitries that regulate telomeres with other cellular machinery.

## Conclusions

We anticipate continued expansion of the telomere interaction network as more telomere-associated proteins are discovered and studies using ChIP-seq, PICh and QTIP technologies are carried out in a wide range of species, cell lines, tissues and in cells with different telomere status. TeloPIN will continue to serve as an important repository with timely update of the newest telomeric protein–DNA–RNA interaction data and better integration and comparison of diverse interaction datasets. We will also incorporate data regarding telomeric protein disease mutants to expand the database, provide more functionally relevant information and ultimately achieve comprehensive understanding of the telomere regulatory network and its role in the development and progression of diseases.

## Supplementary Data

Supplementary data are available at *Database* Online.
